# Depolymerization and Etching of Poly(lactic acid) via TiCl_4_ Vapor Phase Infiltration

**DOI:** 10.1021/acs.jpcc.4c04986

**Published:** 2024-11-13

**Authors:** Shuaib A. Balogun, Mark. D. Losego

**Affiliations:** School of Materials Science and Engineering, Georgia Institute of Technology, Atlanta, Georgia 30318, United States

## Abstract

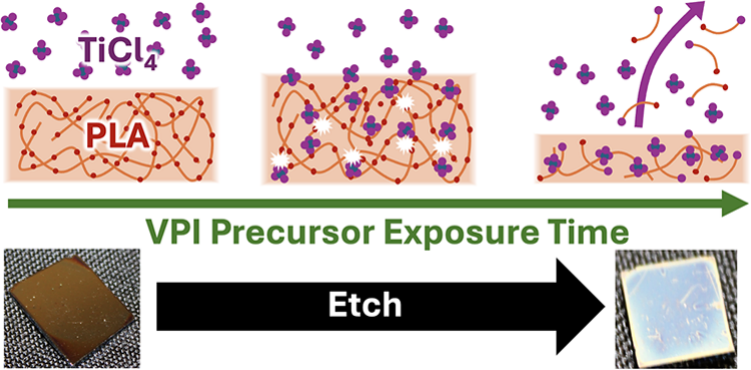

This study investigates the use of TiCl_4_ vapor phase infiltration (VPI) to cleave ester groups in the main chain of a polymer and drive depolymerization and film etching. Prior investigations have demonstrated that the infiltration of TiCl_4_ into PMMA results in dealkylation of its ester bond, cleaving its side groups. This study investigates the VPI of TiCl_4_ into poly(lactic acid), which is a prototypical polymer with an ester group in its main chain. Utilizing *in situ* quartz crystal microbalance (QCM) measurements and spectroscopic ellipsometry, PLA is observed to depolymerize readily at 135 °C with extended TiCl_4_ precursor exposure, resulting in significant thickness and mass reduction, whereas at 90 °C, depolymerization is significantly slower and etching is negligible. Utilizing Fourier transform infrared spectroscopy (FTIR), X-ray photoelectron spectroscopy (XPS), and a residual gas analyzer (RGA), dealkylation is shown to be the primary depolymerization mechanism. FTIR and XPS analyses reveal the consumption of carbonyl and methoxy groups and the emergence of hydroxyl, chlorine, and titanium moieties. *In situ* RGA measurements provide further insights into the byproducts formed during the TiCl_4_ and water exposure steps, indicating that the depolymerized components undergo further breakdown into other substances. Residuals left after 135 °C TiCl_4_ VPI are easily removed with a 0.1 M HCl aqueous solution. These findings highlight the expanding functionality of VPI, revealing its capability as both an additive and subtractive process and suggesting its broader applications.

## Introduction

Vapor phase infiltration occurs when a polymer is exposed to a vapor phase precursor, usually an inorganic, that can sorb into and become entrapped inside the polymer. Entrapment can occur by (1) a chemical reaction between the precursor and the polymer functional group or (2) adducting of the precursor to the polymer’s functional group.^1−5^ Once entrapped, a coreactant (e.g., an oxidant) can be introduced to react with the initial precursor and form a “final” product. When the initial precursor is a metalorganic or metal halide and the coreactant is water or oxygen, the resulting product is often an oxide, hydroxide, or oxyhydroxide, which subsequently remains entrapped in the polymer converting the polymer into an organic–inorganic hybrid material with properties that differ from the pure polymer.

The chemical reaction between a sorbed inorganic precursor and a polymer depends on the chemistry of each and the processing temperature. For example, in the TMA/PMMA system, a reversible adduct forms between the TMA precursor and the carbonyl functional groups of PMMA below 100 °C. Above this temperature, a reaction between TMA and the carbonyl groups leads to the formation of C–O–Al bonds, linking the organic and inorganic components.^1,4,6^ In contrast, for the TiCl_4_/PMMA system, a primary chemical bond occurs between the titanium and methoxy oxygen via a concerted dealkylation reaction between the TiCl_4_ and the ester group.^7^ This reaction results in the loss of a byproduct during the precursor exposure. In prior work, we demonstrated that TiCl_4_ infiltration into PMMA occurs via a reaction-limited mechanism.^7,8^ Unlike TMA infiltration, TiCl_4_ infiltrates uniformly, albeit more slowly, within the entire depth of the PMMA film, i.e., the TiCl_4_ rapidly diffuses into the PMMA, but entrapment is limited by the reaction rate.^7−9^

VPI is also of interest for creating metal-oxide nanostructures. This application of VPI has been enabled by the understanding of reaction and diffusion interplay upon precursor exposure to polymers.^10−20^ For instance, poly(styrene-*block*-4-vinylpyridine) (PS-*b*-P4VP) and poly(styrene-*block*-methyl methacrylate) (PS-*b*-PMMA) are frequently used due to many precursors’ preferences to become entrapped in the P4VP or PMMA blocks but not the PS blocks.^10−13,20,21^ Subsequently, the polymer is removed via plasma etching or oxidative annealing, resulting in an inorganic nanostructure templated by the original block copolymer morphology. Finding new polymer and infiltration chemistries that lead to high inorganic content is of continued interest for related applications.

Poly(lactic acid) (PLA) is a biodegradable polymer that contains an ester functional group in the backbone. The structure of PLA results in a relatively low glass transition temperature (∼65 °C) but a high melting temperature (ca. 150–180 °C), making it suitable for VPI processes.^22^ In fact, PLA has successfully been infiltrated with TMA, where it shows that the carbonyl is fully consumed by the TMA precursor upon subsequent reaction with a water dose.^23^

TiCl_4_ into PLA is yet to be studied. However, insights gained from the TiCl_4_/PMMA system, lead us to hypothesize that TiCl_4_ VPI should cleave the ester groups in the main chain of this polymer’s backbone. Thus, TiCl_4_ VPI may be used as a way to depolymerize or even etch PLA polymers. Upon etching, a titanium oxide type of residue may also be left behind that could be useful in polymer-templating inorganic nanostructures.

To test our hypothesis, PLA was infiltrated with TiCl_4_ at various process temperatures and precursor exposure times and a combination of *in situ* and *ex situ* analyses are used to confirm depolymerization and etching and give some insight into the chemical byproducts.

## Methods

### Materials

Poly(dl-lactic acid) powder with a molecular weight of 20 kDa was sourced from PolySciences, Inc. A 5 wt % solution of this PLA in 99.8% pure toluene (Sigma-Aldrich) was spun-cast onto silicon substrates at 3000 rpm for 30 s to produce films of about 200 nm thickness. For thicker films, a 15 wt % PLA solution in the same toluene was spun-cast at 6000 rpm for 60 s, yielding films of approximately 1 μm thickness. Polymer powder for RGA experiments were prepared by placing this same PLA powder in a heated vacuum oven for 24 h prior to infiltration.

### Vapor Phase Infiltration

PLA films were infiltrated in a custom-built reactor having a 28 L chamber and operated with decision-tree-based control software.^24^ PLA was infiltrated at varying temperatures from 70 to 135 °C with TiCl_4_. The TiCl_4_ precursor was infiltrated with overpressures of ∼1 Torr. All pressures in the reaction chamber were measured with a Baratron capacitance manometer. All VPI processes used a single precursor-*co*-reactant cycle, static hold scheme. The general process sequence was as follows: (1) ultrahigh-purity N_2_ gas was flowed into the reactor to purge the system for 5 min, (2) the system was pumped down to base vacuum (30 mTorr) for an hour for full removal of water, (3) the chamber was isolated, (4) the TiCl_4_ precursor valve, which is connected directly to the chamber, was opened for 5 s to reach a vapor pressure of about 1 Torr TiCl_4_, (5) the TiCl_4_ was then held in the chamber for between 1 and 48 h, (6) the system was then pumped to base vacuum for 5 min, (7) the water coreactant valve, which is also connected directly to the chamber, was opened for 1 s to give a vapor pressure of 1.8 Torr in the chamber, and, (8) the water was held in the chamber for 1 h before purging the system for 60 s and venting to atmosphere.^7,8^

### Spectroscopic Ellipsometry

Film thicknesses were quantified using an α-SE spectroscopic ellipsometer manufactured by Woollam, utilizing a measurement angle of 70° within a spectral range spanning from 340 to 900 nm. Measurements were conducted both before and after infiltration to determine the initial and final film thickness. To derive the film thickness, a Cauchy model was applied. The Cauchy coefficients, denoted as A and B, along with the thickness were subsequently fitted to the data. This film layer was modeled with 2.3 nm native SiO_2_ layer on a silicon substrate.

### *In Situ* Quartz Crystal Microbalance (QCM) Gravimetry Measurements

QCM experiments were performed in a hot-walled custom-built VPI reactor described elsewhere.^9,25^ The QCM used is a Phoenix high-temperature, film-thickness sensor, PC-based system purchased from Colnatec. A polished gold 6 MHz RC quartz crystal was used as the substrate for PLA. The crystal and the surrounding walls were heated to the desired infiltration temperature (90 or 135 °C) under vacuum and flowing nitrogen to determine its baseline resonance frequency. The PLA was spun-cast using the same method as that above. The coated crystal was then placed in the reactor and heated to the desired infiltration temperature (90 or 135 °C) under vacuum and flowing nitrogen to determine the resonance frequency with just the bare polymer. VPI was conducted using similar methods described above except for three key differences: (1) the TiCl_4_ precursor valve, which is connected directly to the chamber, was opened for 0.5 s to reach a vapor pressure of about 3.2 Torr TiCl_4_ due to the smaller volume of this reactor and (2) the film is pumped to base vacuum for 24 h between the TiCl_4_ dose and the water dose to ensure all unreacted precursor and byproducts have sufficient time to escape the polymer. Crystal frequency was recorded every second during this time and was exported and converted to mass via the Sauerbrey equation.^26^

### X-ray Photoelectron Spectroscopy (XPS)

XPS was performed using the Nexsa G2 Surface Analysis System with a monochromatic Al–Kα X-ray source (1486.6 eV) with a 60° incident angle and a 90° emission collection geometry. Survey scans were conducted at a pass energy of 200 eV and for binding energies from −10 to +1350 eV. For the elemental analysis, the following elements at the following binding energies were collected: Ti 2p (448–475 eV), O 1s (525–545 eV), C 1s (279–298 eV), Cl 2p (190–210 eV), and Si 2p (95–110 eV). In scenarios in which both chemical peak and elemental compositions were required, a cluster ion gun (MAGCIS dual-mode ion source) was utilized for etching. The procedure involved a raster size of 400 μm × 400 μm and an ion gun voltage of 8000 eV with a cluster size of 150 amu, along with an active flood gun. At each etch level, elemental analysis and survey scans were carried out using “snapshots” to provide a representative analysis of the elemental chemical peaks of the thin film. For these analyses, Shirley background subtraction was applied to determine the atomic percentage of titanium, while other atomic percentages were determined by using a simple background subtraction technique.

### Residual Gas Analyzer

An EXtorr XT Series Residual Gas Analyzer operated on a PF70 turbomolecular vacuum pump is attached to the hot-walled custom-built VPI reactor described above via a capillary tube to sample the reaction atmosphere. The mass-to-charge ratio (*m*/*z*) was determined with a scan speed of 48 scans/s from 1 to 200 atomic mass units. The gas atmosphere was sampled by (1) opening a valve that transmits the gas in the reactor to the RGA and (2) turning on the filament inside the RGA and analyzing the gas atmosphere. Prior to the *in situ* experiment for mass spectrometry analysis, the gas line leading to the residual gas analyzer (RGA) was passivated with a dose of the TiCl_4_ precursor. PLA powders were used instead of thin films to increase the signal intensity of byproduct formation, making it easier to identify these species within the mass spectra. 0.4 g of PLA powder was measured and used for all RGA experiments.

### Fourier Transform Infrared Spectroscopy

Attenuated total reflectance Fourier transform infrared (ATR-FTIR) spectroscopy was performed on 1 μm PLA films on double-sided polished, low-conductivity silicon. The spectra were collected on a Thermo Scientific Nicolet iS5 FTIR spectrometer with an iD7 ATR accessory and a diamond crystal. Spectra were collected with a resolution of 4 cm^–1^ and are the average of 64 scans.

### Dissolution Experiments

Dissolution experiments were performed by placing 10 mL of solvent in a glass vial. Subsequently, the infiltrated thin films were placed in the solvents for 24 h to ensure thorough dissolution of the products. Films were removed from solution at various time intervals and placed in a fume hood for 5 min to dry, and then thickness was measured via ellipsometry to determine the extent of film dissolution. The surface chemistry of the films postdissolution was analyzed using XPS to gain further insights into the chemical changes occurring on the film surfaces. The solvents selected for these experiments included toluene, water, ethanol, and 0.1 M hydrochloric acid, chosen for their varied solvating capabilities for the different film constituents.

## Results and Discussion

We have previously established that TiCl_4_ reacts with the ester functional group of PMMA, breaking the methoxy bond to release the methyl group as a CH_3_Cl byproduct and leaving a C–O–TiCl_3_ linkage.^7^ To further illustrate the ability of metal halides to cleave ester linkages, this paper explores the VPI of TiCl_4_ into poly(lactic acid) (PLA), which contains ester linkages in the polymer backbone. We hypothesize that TiCl_4_ VPI should depolymerize PLA and potentially etch the polymer if the residual byproducts are sufficiently volatile at the VPI process temperatures.

**Chart 1:**
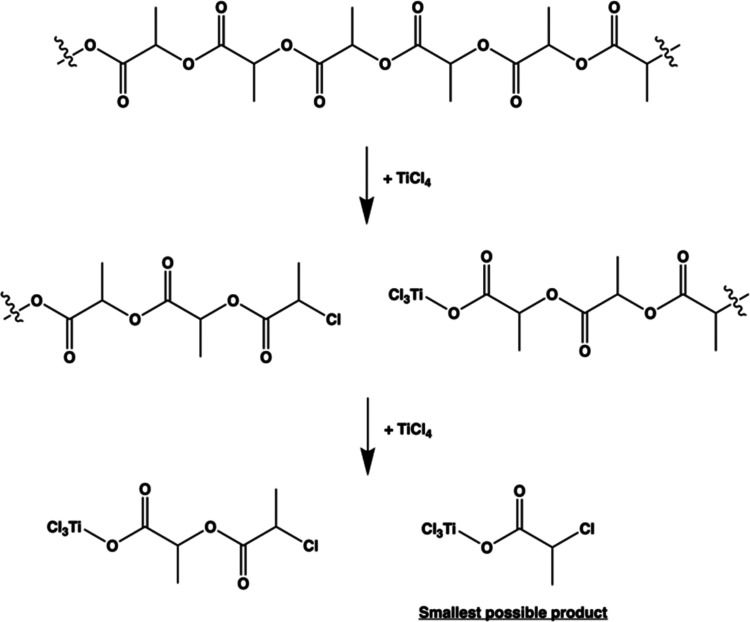
Hypothesized Breakdown of PLA Based on the TiCl_4_ Performing a Dealkylation Reaction at the Ester Functional Group

**Chart 2:**
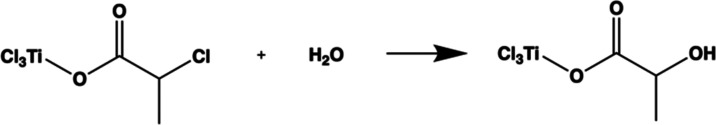
Hypothesized Hydrolysis of Organochloride Moieties formed via Chart 1 Resulting in Hydroxyls

Chart 1 depicts the hypothesized reaction mechanism for the depolymerization of PLA upon exposure to TiCl_4_. This mechanism includes scission of the polymer backbone at random ester group sites, thereby leading to products of multiple sizes. The smallest possible product is shown at right in the bottom step. Chart 2 depicts how this smallest possible product or similar organochloride oligomers could be hydrolyzed during the water exposure step to form alcohols, a process that will be discussed later in this study. It is important to note that water exposure could potentially also react with the O-TiCl_3_ moiety but as observed in previous work the TiCl_3_ could continue acting as a Lewis acid causing more polymer cleavage to occur.^7^

**Figure 1:**
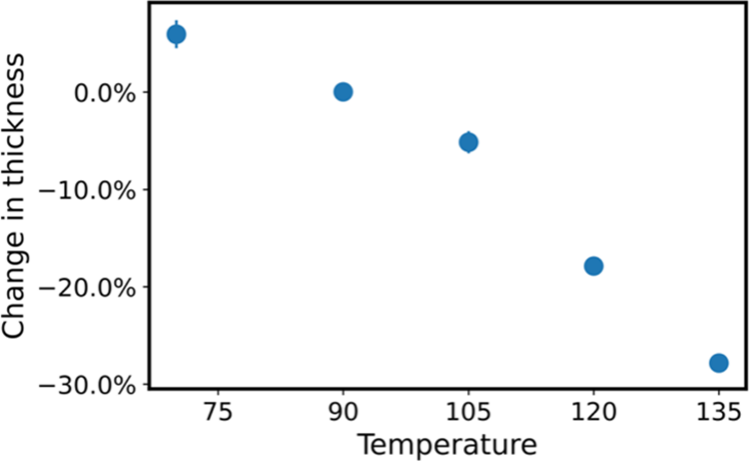
Change in thickness derived from spectroscopic ellipsometry of 200 nm PLA thin films infiltrated with TiCl_4_ at various temperatures (70–135 °C) with 12 h of precursor exposure time. Change in thickness is calculated by determining the thickness before and after infiltration.

To test this hypothesis, we infiltrated PLA thin films of 200 nm thickness with TiCl_4_ for 12 h at varying process temperatures. Figure 1 plots the percent change in thickness for these 200 nm PLA films after VPI processing at varying temperatures (70–135 °C). Above 90 °C we see a reduction in film thickness that is consistent with the hypothesized depolymerization and etching process expected for this chemistry. However, below 90 °C, films increase in thickness. This result suggests that the depolymerization and volatilization are temperature-dependent, and at lower process temperatures, TiCl_4_ is likely infiltrating (hence the increased film thickness), but depolymerization and/or volatilization are incomplete. To better understand these phenomena, we proceed to investigate more carefully the TiCl_4_ infiltration processes at the temperature extremes of 135 and 90 °C, respectively.

### TiCl_4_ Infiltration at 135 °C

**Figure 2:**
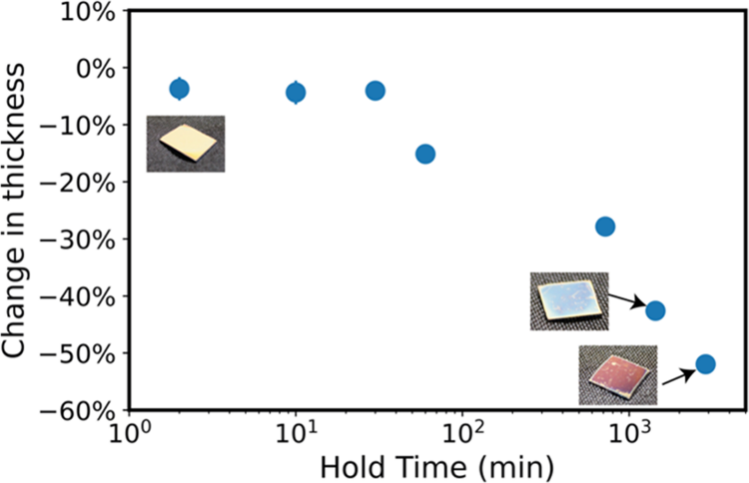
Percent change in film thickness before and after infiltration as determined by spectroscopic ellipsometry for 200 nm PLA thin films infiltrated with TiCl_4_ at 135 °C at varying precursor exposure times (0–48 h). Photographs show the visual change in the film thickness.

To better understand the process kinetics at 135 °C, Figure 2 presents the change in thickness of 200 nm PLA films infiltrated with TiCl_4_ at 135 °C at varying precursor exposure times (0–48 h). We can observe a decrease in the thickness of PLA at all exposure times even within ∼10 min. By 48 h of exposure time, the film is reduced to 50% of its original thickness. While the etching process appears to slow with time (log scale time), it does not appear to have reached a saturation point by 48 h. This change in thickness is also very apparent visually, as indicated by the photographs of the films in the insets of Figure 2. Figure S1 shows the change in thickness and the corresponding change in the refractive index of the thin films.

**Figure 3:**
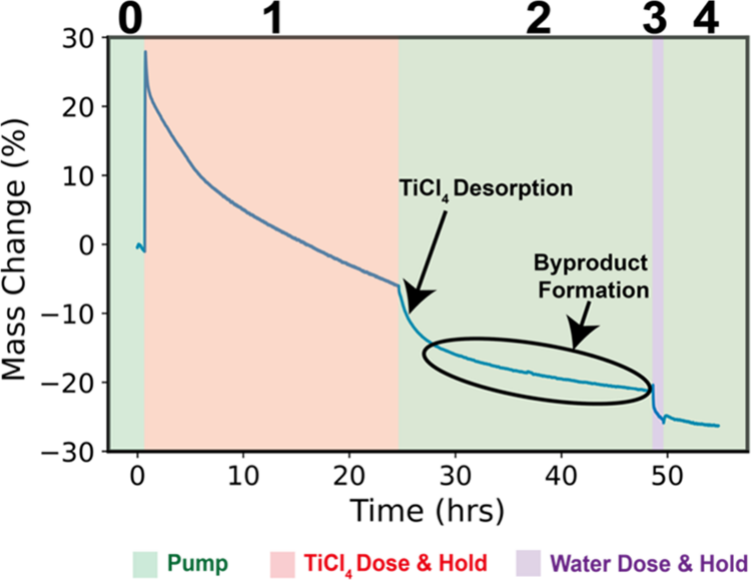
*In situ* quartz crystal microbalance gravimetry of TiCl_4_ infiltration into PLA at 135 °C with 24 h of precursor exposure time. The plot is separated into five temporal regimes: (0) preinfiltration pumping (vacuum base pressure), (1) TiCl_4_ exposure (3.2 Torr), (2) TiCl_4_ removal via vacuum pumping, (3) water exposure (1 h), and (4) water removal via vacuum pumping. The mass change is normalized to the original mass of the polymer (24.5 μg) to provide a percentage of mass added to the polymer via infiltration. The mass uptake is the additional mass gained (or lost) from this starting polymer weight, indicated as a percentage. All masses are calculated from the Sauerbrey equation.

To ensure that the thickness loss is truly a result of the TiCl_4_ exposure step, *in situ* QCM is used to monitor the mass of the polymer during infiltration. Figure 3 presents a quartz crystal microbalance (QCM) gravimetry analysis of the TiCl_4_–PLA VPI process collected at 135 °C. While an in-depth analysis of this data is beyond the scope of this paper, a qualitative assessment provides further evidence supporting the mechanism of thickness loss observed in Figure 2.

The QCM data in Figure 3 are separated into 5 regions: (0) preinfiltration pumping, (1) TiCl_4_ dose (0.5 s) and TiCl_4_ exposure (24 h), (2) TiCl_4_ removal via vacuum pumping (24 h), (3) water exposure (1 h), and (4) water removal via vacuum pumping. In region 1 of Figure 3, the observed rapid rise in mass uptake is consistent with the sorption of TiCl_4_ into PLA. This sorption increases the polymer’s weight by ∼30% (7.35 μg) within 5 min of TiCl_4_ exposure. Beyond 5 min of TiCl_4_ exposure, mass is lost and this mass loss is continuous over the time period explored (24 h). Around 18 h of TiCl_4_ exposure, the QCM measured mass drops below zero, indicating the film’s mass is now less than the original film, which can only happen if mass from the film is being volatilized. Thus, this result gives us high confidence that we are not just observing out-diffusion of our precursor but rather a significant loss of organic species being removed (etched) from the original polymer film. Note that these QCM data also suggest that etching will continue to proceed beyond 24 h. Spectroscopic ellipsometry was used to determine the change in thickness of the PLA thin film on the QCM crystal in Figure 3. The measured thickness loss was 42% of the original film thickness. This is approximately similar to the observed thickness loss observed for thin films exposed to the same process conditions in Figure 2.

In region 2 of Figure 3, the system is subjected to vacuum pumping, and a more rapid mass loss is observed. We attribute the faster mass loss to the desorption of dissolved but unreacted TiCl_4_ species since the TiCl_4_ overpressure has been removed. However, beyond about 30 h, a linear decrease in the mass persists with a slope similar to that observed near the end of region 1. If this mass loss was only due to TiCl_4_ desorption, then an asymptotic saturation in the removal would be expected. This continued linear decrease in mass suggests the potential continuation of TiCl_4_-driven depolymerization and byproducts removal. This shows that the TiCl_4_ that has already sorbed and is immobilized inside the polymer can continue to react, consistent with our prior report that a single TiCl_4_ species likely reacts with multiple ester groups.^7^

Upon exposure to water in region 3, another abrupt mass loss occurs. This mass loss is suspected to be the reaction of water with residual Ti–Cl or possibly C–Cl bonds (Chart 2) to create hydroxyls and a volatile HCl byproduct. At the end of the final pumping step, the film’s mass is ∼27% lower than its original mass and 57% lower than the maximum mass during infiltration.

**Figure 4:**
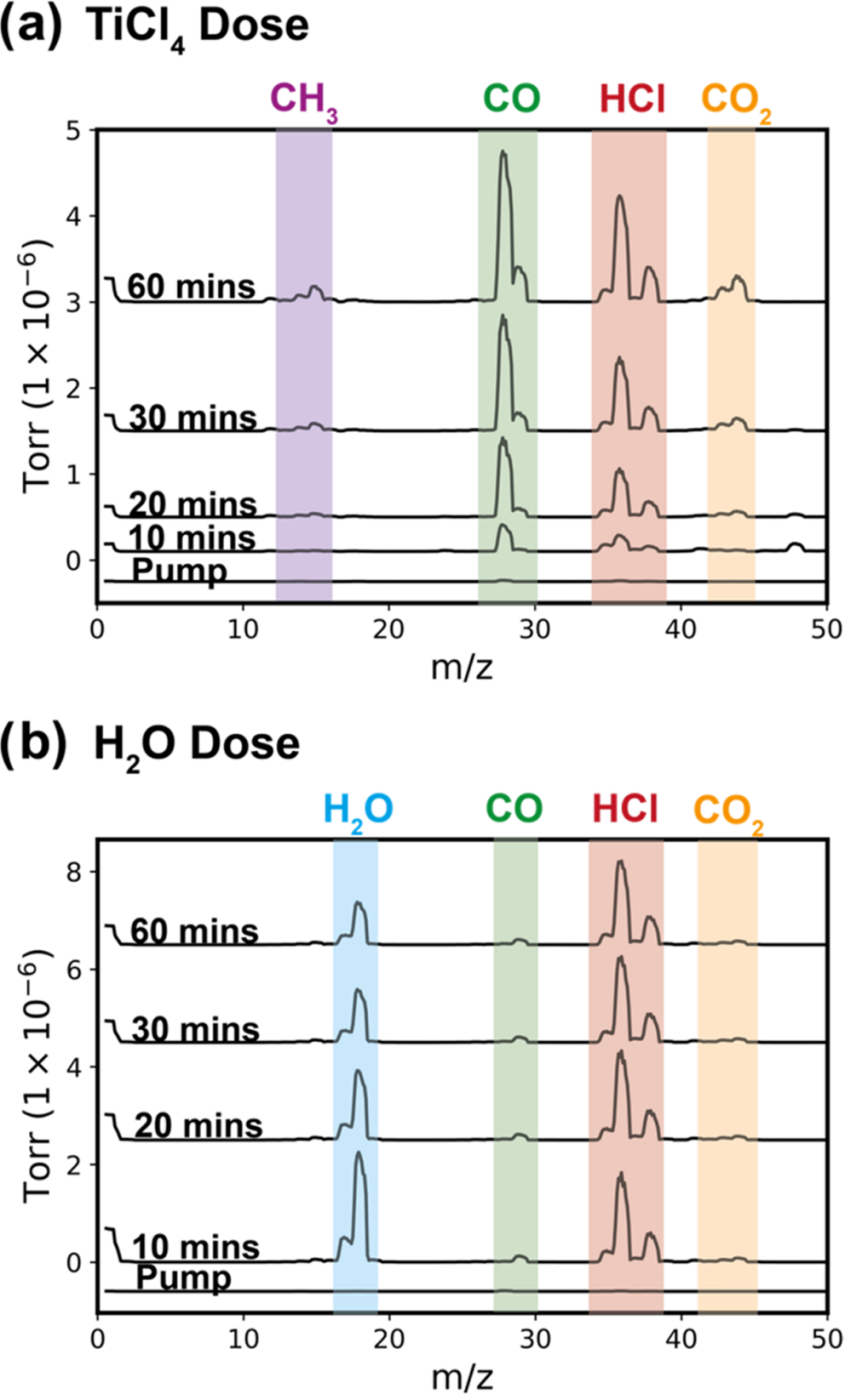
RGA mass spectra of the gas species above PLA powder exposed to (a) TiCl_4_ and (b) H_2_O at 135 °C processing temperature at different exposure times. The mass spectra data are segmented into distinct time intervals: 0 (preinfiltration vacuum pumping), 10, 20, 30, and 60 min of precursor or coreactant exposure. Evolved peaks are highlighted in different colors on the spectra and labeled respective to the identified compound.

To explore the chemical reaction occurring for TiCl_4_ infiltration into PLA at 135 °C, the reaction atmosphere is sampled with a residual gas analyzer within regions 1 and 3 of Figure 3. Figure 4 shows the mass spectra collected from these atmospheres during both the (a) TiCl_4_ and (b) H_2_O exposure steps. Note that PLA powders are used in the chamber for these measurements instead of PLA films to increase the concentration of byproducts to detectable levels at shorter times. During the preinfiltration vacuum pumping step, no peaks are present due to the chamber being actively pumped of residual gases.

In Figure 4a, as TiCl_4_ exposure time increases, new peaks emerge at *m*/*z* values of approximately 15, 28, 36, and 44. These peaks most likely correspond to fragments of methyl (CH_3_), carbon monoxide (CO), hydrochloric acid (HCl), and carbon dioxide (CO_2_), indicating a progression in the chemical reactions within the chamber. To identify the byproducts formed, we analyzed the mass spectra for the emergence of peaks at higher *m*/*z* values, particularly those that might indicate the presence of titanium compounds (around *m*/*z* of 47). The analysis at 60 min revealed no discernible peaks with a *m*/*z* above 45, confirming the absence of volatile titanium compound byproducts. This observation also suggests a lack of chlorine-based byproducts other than HCl. Furthermore, the analysis did not detect any peak pairs separated by 2 *m*/*z* units with a 3:1 ratio, a characteristic signature of chlorine-based compounds. Thus, this spectral evaluation suggests that the volatile byproducts during TiCl_4_ exposure are primarily HCl and some organics.

**Chart 3:**
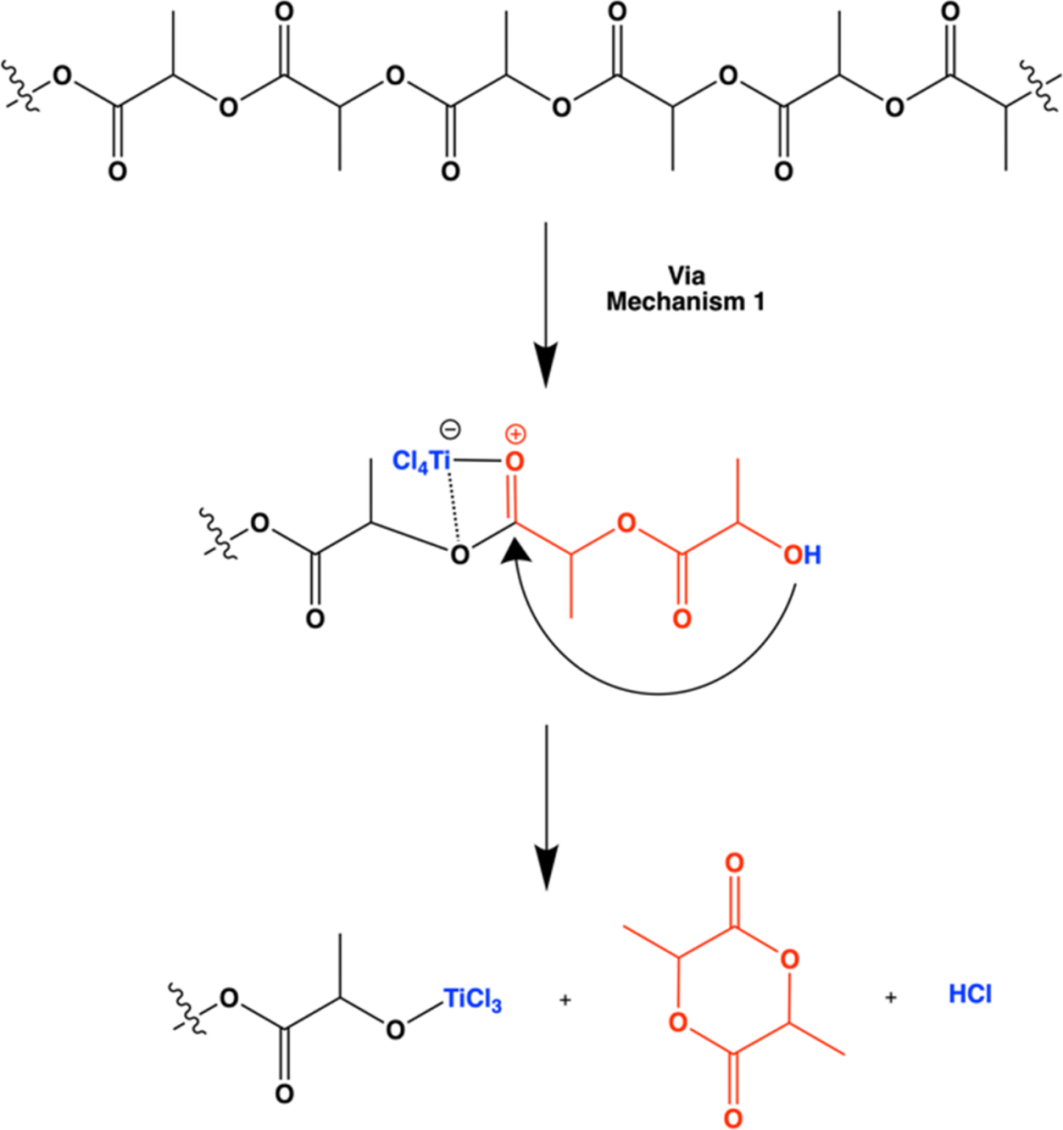
Lactide Formation due to Depolymerization of PLA in the Presence of TiCl_4_ and OH End Groups

Initially, like in Chart 1, we posited that the organic byproducts may be a derivative of lactic acid; however, lactide formation is also a possibility. Chart 3 depicts how depolymerization could lead to the formation of lactide. Note this scheme includes hydroxyl groups that could originate from the polymer’s end group caps or via hydrolysis of products with a chloride group created in Chart 1. These lactides are fully organic and thus could explain the purely organic byproducts being detected. At 135 °C, lactic acid and lactide should be volatile (lactic acid is above its boiling point and lactide has a vapor pressure of 0.7 Torr) and their characteristic peaks should be observed in the mass spectra at 45 and 56, respectively, but neither are observed in our RGA spectra. Thus, it appears that these organics are potentially decomposing further to fully volatilize. Such subsequent decomposition is consistent with prior findings that lactic acid and lactide can degrade into acetaldehyde, carbon dioxide, and methane at elevated temperatures, corresponding to the observed peaks at *m*/*z* ∼15, ∼28, and ∼44.^27,28^ Additionally, from these studies it is possible for lactic acid to decompose to produce water.^27,28^ Although the process temperature is less than that reported in other studies (∼300 °C), the presence of other compounds such as HCl and TiCl_4_ may facilitate this decomposition at lower temperatures.

The formation of HCl during the TiCl_4_ hold step is unexpected. It indicates a hydrolysis reaction during the TiCl_4_ hold step. This hydrolysis could be the result of: (1) water in the chamber, (2) hydroxyls present in the polymer or in the byproduct formed by the polymer (lactic acid/lactide), or (3) water generation from the breakdown of lactic acid/lactide at high temperatures in the presence of TiCl_4_, which can serve as a catalyst. Chamber water content should be quite low given that a prepassivation step is used to remove water from the chamber walls and prepurge steps are extensive. The prepassivation step involves dosing and removal of TiCl_4_ into the reaction chamber and RGA prior to infiltration to remove all residual water. The effects of a prepassivation step on the mass spectra of a TiCl_4_ dose can be observed in Figure S2. Thus, we cannot make any conclusive claims about the source of water for these apparent hydrolysis reactions during the TiCl_4_ exposure step.

Figure 4b presents the mass spectra for the reactor atmosphere during the water exposure step. Two primary peaks are observed at *m*/*z* values of approximately 18 and 36. These peaks correspond to fragments of H_2_O (water) and HCl (hydrochloric acid). As exposure time increases, the H_2_O decreases, while the HCl peak remains approximately constant. This decrease in the water signal is further evidence that no significant water leaks are occurring in this chamber. The H_2_O decrease can be attributed to (1) reaction between the H_2_O and sorbed TiCl_4_ species resulting in HCl byproduct formation or (2) H_2_O sticking to the walls or (3) sorbing to the residual PLA powder after TiCl_4_ exposure. The continued but nearly constant presence of HCl in the vapor phase is consistent with the rapid, nearly immediate mass loss observed in the QCM at the start of the water exposure step. HCl is formed initially, but then no further reaction takes place

**Figure 5:**
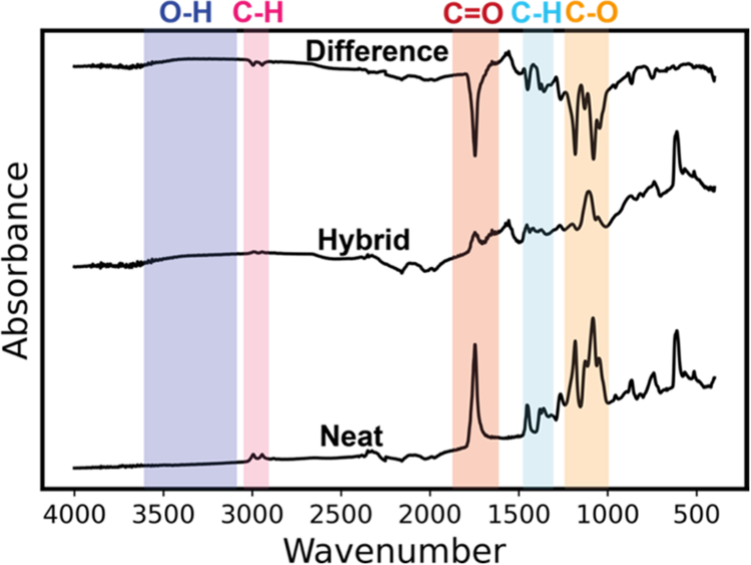
FTIR showing PLA infiltrated with TiCl_4_ at 135 °C. The FTIR plot shows the individual spectra for neat PLA (bottom), PLA infiltrated with TiCl_4_ at 135 °C (middle), and the difference spectrum (top).

To better understand the final chemical structure of the resulting PLA-TiO_*x*_ hybrid material, these materials were studied with *ex situ* FTIR and XPS. Figure 5 plots the IR spectra of neat PLA and PLA infiltrated with TiCl_4_ at 135 °C for 24 h of exposure time (“Hybrid”) and the resulting difference spectrum. Neat PLA has characteristic peaks that represent C–O stretching at 1080–1180 cm^–1^, C–H bending at 1300–1470 cm^–1^, C=O stretching at ∼1746 cm^–1^, and −CH stretching at 2900–3000 cm^–1^. The hybrid PLA-TiO_*x*_ shows a significant reduction in all characteristic peaks, indicating that these bonds are either consumed or removed from the film. Based on the reduction in film thickness, mass loss observed in *in situ* QCM, and carbon loss detected via RGA, we conclude that much of this reduction is a consequence of organic etching. However, not all carbon is removed, with residual absorptions for C–H, C=O, and C–O. Notably, a hydroxyl stretch emerges at 3300–3500 cm^–1^. These hydroxyls further corroborate the proposed water hydrolysis of residual metal chloride or carbon chloride bonds proposed from the mass loss observed in region 3 of Figure 3 and the HCl byproducts detected in Figure 4b.

**Figure 6:**
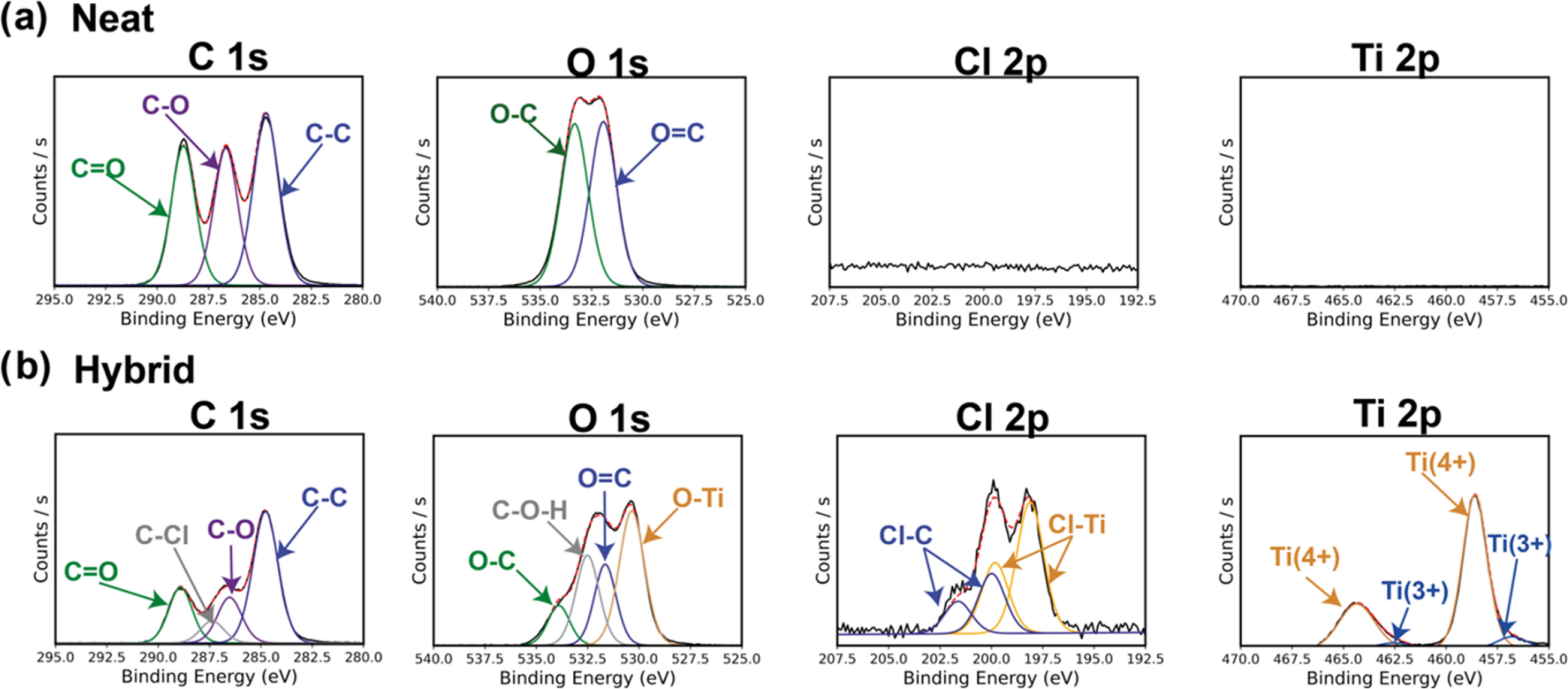
XPS spectra for (a) neat PLA and (b) PLA infiltrated with TiCl_4_ + H_2_O at 135 °C. The C 1s, O 1s, Cl 2p, and Ti 2p spectra are shown for each PLA and PLA-TiO_*x*_ hybrid film. Emission intensity axes (abscissas) are kept constant down each column (i.e., for each element). Deconvolutions are labeled for the C, O, Cl, and Ti spectra.

Figure 6 presents XPS spectra for a PLA film infiltrated with TiCl_4_ at 135 °C for (a) 0 (neat PLA) and (b) 24 h. Note that these are collected from the film’s surface. Surface scans were used because the surface is assumed to be the most saturated portion of the infiltrated polymer.^2,3,9^Figure 6a shows the XPS of a neat PLA film. As expected for PLA’s stoichiometry, the C 1s spectrum has nearly equal concentrations of C–C (284.8 eV), C–O (286.8 eV), and C=O (288.8 eV) chemical states. Similarly, the spectrum of the O 1s shows equal amounts of methyl-oxy (C–O, 533.6 eV) and carbonyl (C=O, 531.8 eV) states. No peaks are present at the Ti 2p and Cl 2p energies. Figure 6b presents the same spectral edges after the infiltration. Now, in the C 1s spectrum, a C–Cl peak emerges at 287.3 eV and the C–O and C=O emissions appear reduced relative to the C–C emission. This latter observation (high C–C emission) is thought to be an artifact of adventitious carbon adsorbed to the surface. Upon cluster ion etching in the XPS chamber, which is expected to nondestructively remove organic material,^29^ we find the C–C, C–O, and C=O emissions to return to nearly equal intensities (Figure S3), suggesting that the material is actually being etched relatively stoichiometrically by TiCl_4_ with some residual adventitious carbon on the surface. Upon infiltration, the O 1s spectra show the emergence of a O–Ti peak at ∼530.5 eV. Concurrently, the C–O peak is reduced relative to the C=O peak, and a C–O–H (hydroxyl) peak appears at 532.7 eV. This data suggests that the ratio of C–O to C=O bonds is decreasing with the infiltration reaction, consistent with Chart 1 in which the ester bond breaks and the C–O–C linkage is replaced with a M–O-C linkage. If we examine the smallest final product in Chart 1, further insights can be gained. As depicted in Chart 2, the chloride moiety could hydrolyze during the water dose, leading to hydroxyl functionality. Such hydroxyls are observed in both the XPS spectra of Figure 6b and the FTIR spectra of Figure 5. The Cl 2p spectra provide further evidence for Charts 1 and 2. The Cl 2p is expected to be a doublet, but in this spectrum, two doublets are observed and can be assigned to Ti–Cl at 198.5 and 199.5 eV and C–Cl at 200 and 201 eV. This C–Cl emission is consistent with the C–Cl peak observed in the C 1s spectrum and provides further evidence that organochloride functional groups exist after VPI, as proposed in Chart 1. Finally, the Ti 2p spectrum shows clear doublets at 458.7 and 464.45 eV, consistent with Ti in its 4+ oxidation state. Ti 2p spectrum has a small doublet at 457 and 462.5 eV consistent with Ti in its 3+ oxidation state but is minimal compared to the 4+ oxidation state.

### TiCl_4_ Infiltration at 90 °C

**Figure 7:**
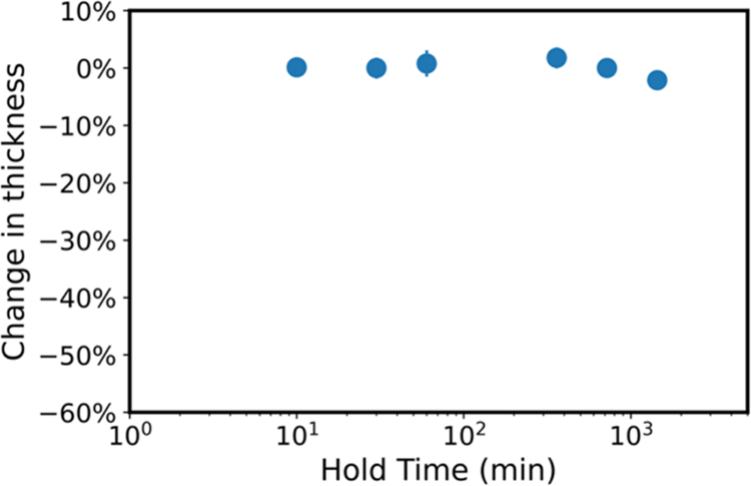
Change in thickness derived from spectroscopic ellipsometry of 200 nm PLA thin films infiltrated with TiCl_4_ at 90 °C at various precursor exposure times (0–24 h). Change in thickness was calculated by determining the thicknesses before and after infiltration.

In this section, characterization techniques similar to those used in the prior section for infiltration at 135 °C are applied to study the process of TiCl_4_ infiltration into PLA at 90 °C. In the Supporting Information we replot the data sets for each of these characterization methods for the 90 and 135 °C process next to each other for easier comparison (Figures S4–S7). However, in general, TiCl_4_ infiltration at 90 °C shows similar chemical mechanisms to infiltration at 135 °C, except that the dealkylation reaction is significantly slower, which leads to less mass loss and nominal film thickness changes. For example, Figure 7 plots the change in thickness of 200 nm PLA thin films upon infiltration at 90 °C at varying precursor exposure times (0–24 h). Unlike the 135 °C process, no significant film loss (or etching) appears to occur at 90 °C.

**Figure 8:**
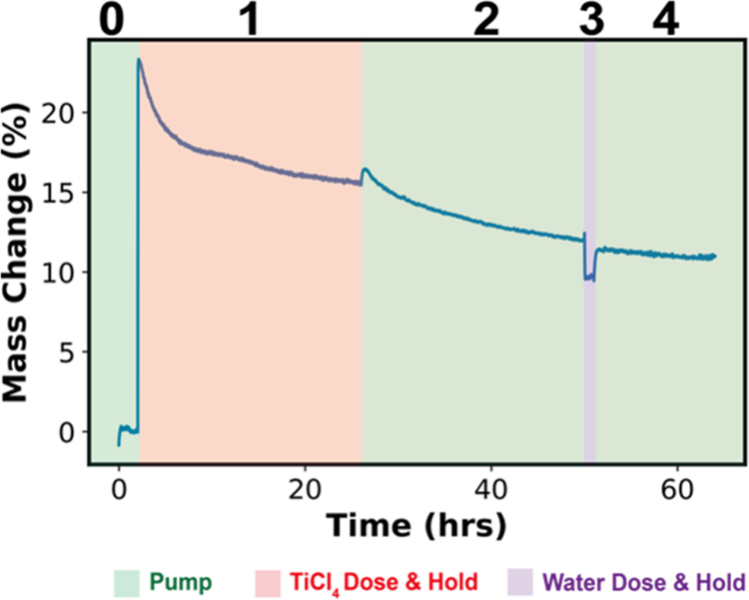
*In situ* QCM gravimetry of TiCl_4_ infiltration into PLA at 90 °C with 24 h of precursor exposure time. The plot is separated into three temporal regimes: (0) preinfiltration pumping (vacuum base pressure), (1) TiCl_4_ exposure (3.2 Torr), (2) TiCl_4_ removal via vacuum pumping, (3) water exposure (1 h), and (4) water removal via vacuum pumping. The mass change is normalized to the original mass of the polymer (29.9 μg) to provide a percentage of mass added to the polymer via infiltration. The mass uptake is the additional mass gained from the starting weight of the polymer indicated in the initial pumping as a percentage. All masses are calculated from the Sauerbrey equation.

This reduction in overall mass loss is further evident in the *in situ* QCM data plotted in Figure 8. This QCM data is again separated into 5 regions: (0) preinfiltration pumping, (1) TiCl_4_ exposure (24 h), (2) TiCl_4_ removal via vacuum pumping (24 h), (3) water exposure (1 h), and (4) water removal via vacuum pumping. At the start of region 1, the mass uptake rises rapidly, consistent with sorption of TiCl_4_ into PLA. The peak mass uptake represents ∼23% of the polymer’s weight (29.9 μg). After 5 min of TiCl_4_ exposure, the mass begins to decrease, albeit not as quickly as observed in the 135 °C process. The decrease in mass continues throughout the TiCl_4_ exposure step and results in a ∼7% (2.09 μg) decrease in the overall mass uptake of the polymer, compared to the 34% (8.34 μg) loss observed at 135 °C. During the vacuum pumping step, mass continues to be lost, but this loss is attributed to out-diffusion of unbound TiCl_4_ precursors and possibly reaction byproducts. Note, though, unlike the 135 °C process, the mass never goes below the original mass of the polymer (i.e., below 0%). Upon exposure to water, the mass drops abruptly, but much of this mass is recovered upon purging of the water vapor. An exact explanation for this behavior is currently not clear, although we suspect it is an artifact due to the pressure change in the chamber upon water dosing. Typically, a frequency adjustment can be performed to determine the real frequency change (mass change), but in this experiment, the frequency adjustment could not be determined. Nonetheless, at the end of infiltration, the final product has a positive mass change that represents ∼12% of the polymer weight (3.59 μg), indicating an overall mass gain. This total mass gain indicates that the mass sorbed from infiltration of the TiCl_4_ is a greater overall mass uptake than any mass loss from the removal of reaction byproducts. Moreover, it is interesting to note that although a modest mass gain is observed (∼12%), no significant change in film thickness is detected (Figure 7), again illustrating that volume expansion and mass change are not always directly correlated in vapor infiltration processes.

**Figure 9:**
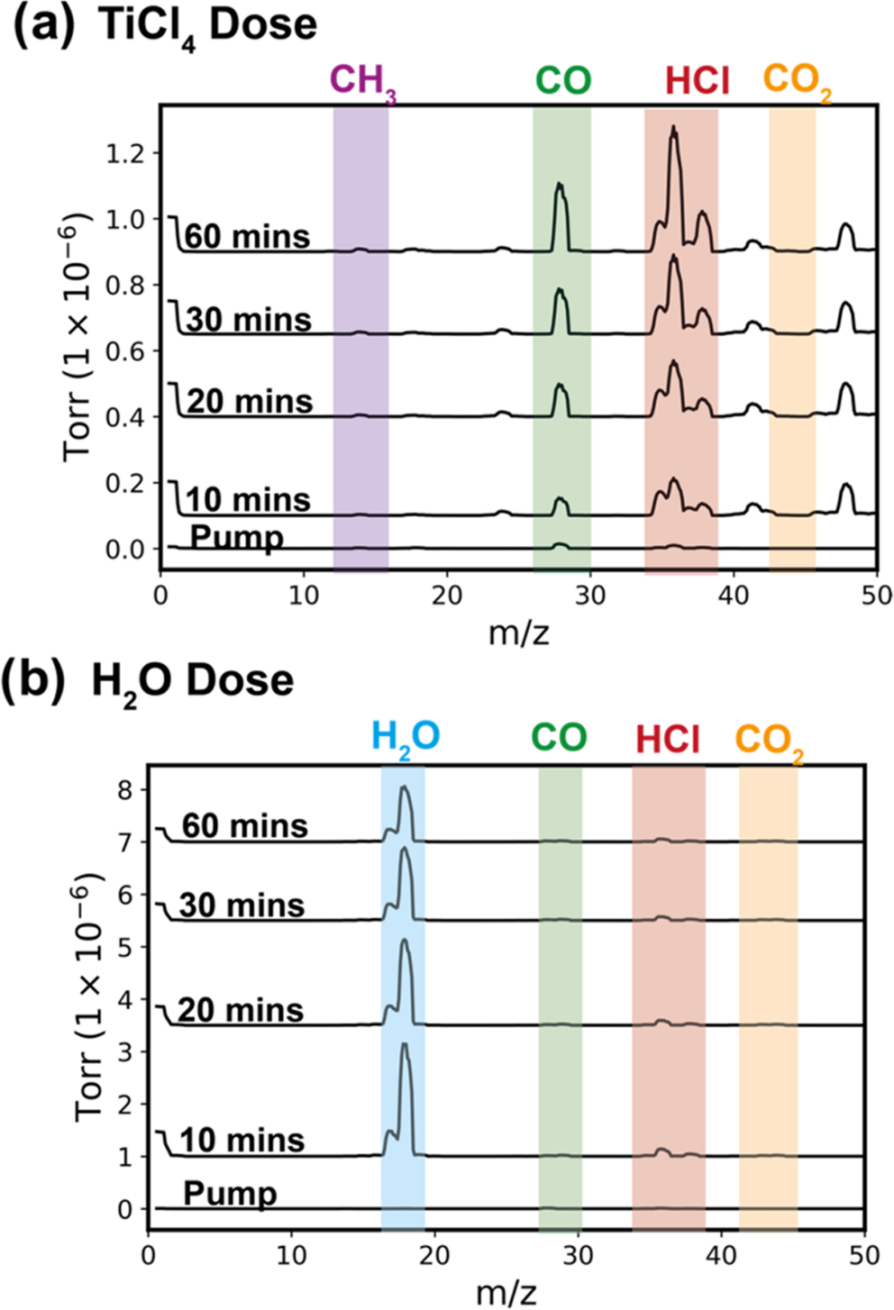
RGA mass spectra of the gas species above PLA powder exposed to (a) TiCl_4_ and (b) H_2_O at 90 °C processing temperature at different exposure times. The mass spectra data is segmented into distinct time intervals: 0 (preinfiltration vacuum pumping), 10, 20, 30, and 60 min of precursor or coreactant exposure. Evolved peaks are highlighted in different colors on the spectra and labeled respective to the identified compound.

To better understand the chemical mechanisms of this 90 °C VPI process, RGA measurements were made during regions 1 and 3 of Figure 8, but note that these measurements were made in separate experiments using PLA powder to increase the concentration of volatile products. Figure 9 plots the relevant mass spectra of the vapor in the reaction chamber when PLA crystals are exposed to (a) TiCl_4_ and (b) H_2_O at 90 °C (full spectra are shown in Figure S4). No signals are detected during the preinfiltration vacuum pumping step.

Like the 135 °C process reported in Figure 4a, Figure 9a shows that new peaks emerge at *m*/*z* values of approximately 15, 28, 36, and 44 upon TiCl_4_ exposure. However, the generation of these peaks is significantly smaller than in Figure 4a. This lower intensity suggests that the formation of byproducts is occurring slower at 90 °C than 135 °C, corroborating the smaller mass loss rates observed by QCM. Upon water exposure (Figure 9b), we can detect the water vapor but little else. At 135 °C (Figure 4b), significant HCl was detected in addition to water during the water exposure. However, HCl generation is minimal at 90 °C, suggesting that minimal TiCl_4_ and C–Cl functionalities are available for water hydrolysis. In addition to the data here, Figure S8 shows the full mass spectrum and Figure S9 shows a comparison between the characteristic peaks of TiCl_4_ during the processes conducted at 90 and 135 °C. One important observation in the full spectrum is that TiCl_4_ is detectable at all exposure times when processed at 90 °C, whereas the TiCl_4_ intensity decreases to zero upon exposure at 135 °C, suggesting that the precursor is fully sorbed/consumed at the higher process temperature, consistent with the more intense reactions observed at the higher temperature.

**Figure 10:**
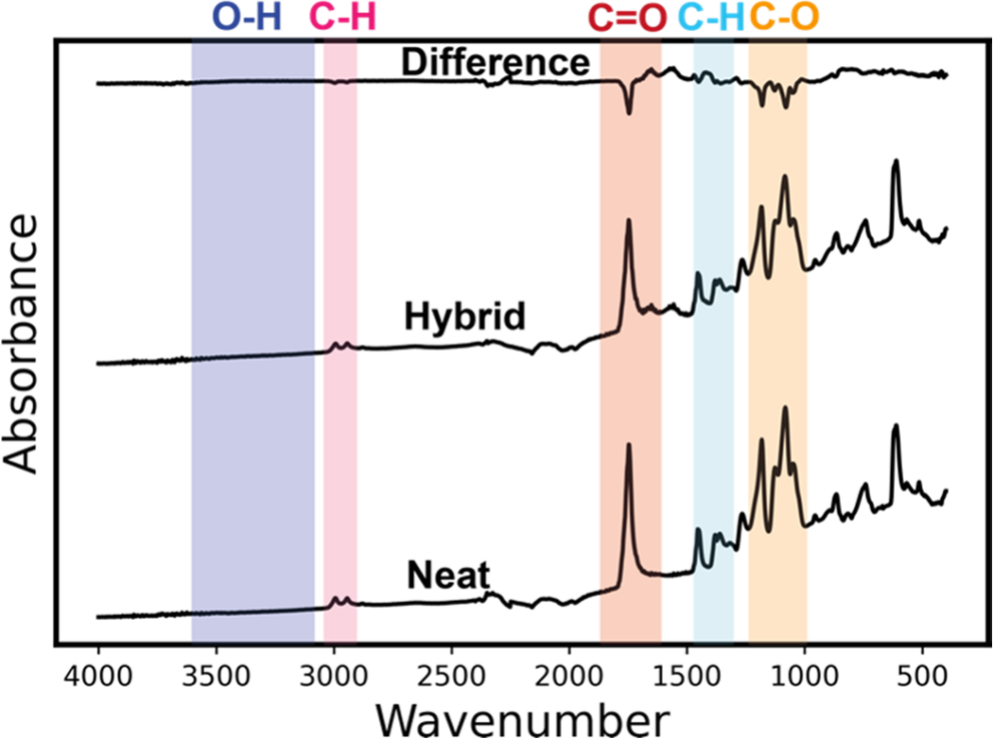
FTIR plot showing the individual spectra for neat PLA (bottom), PLA infiltrated with TiCl_4_ at 90 °C (middle), and the difference spectra (top).

Figure 10 presents *ex situ* IR spectra of neat PLA and PLA infiltrated with TiCl_4_ at 90 °C for 24 h of exposure time (“Hybrid”) and the resulting difference spectra. The IR spectra of the hybrid material show that it is largely unchanged compared to pure PLA. C–H, C=O, and C–O stretches show small decreases in intensity, indicating only minor consumption or interaction between the precursor and the polymer at 90 °C, even though QCM data suggest significant inorganic loading (∼10 wt %).

**Figure 11:**
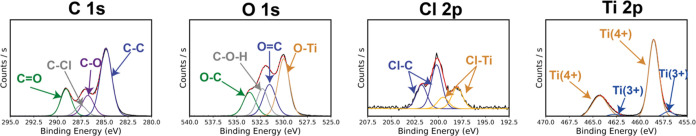
XPS spectra for a PLA film infiltrated with TiCl_4_ + H_2_O at 90 °C. The C 1s, O 1s, Cl 2p, and Ti 2p spectra are shown for the PLA-TiO_*x*_ hybrid film. Emission intensity axes (abscissas) for each element are kept constant with the spectra shown in Figure 6. Deconvolutions are labeled for the C, O, Cl, and Ti spectra.

Figure 11 presents XPS data for PLA films infiltrated with TiCl_4_ at 90 °C for 24 h. These spectra are qualitatively very similar to those measured for infiltration at 135 °C. The C 1s spectrum shows significantly reduced C–O and C=O intensities relative to C–C bonds, but this result appears to again be attributed to the adsorption of adventitious carbon to these surfaces. One possible difference in intensities is noted in the O 1s and Cl 2p spectra. Relative to 135 °C processing, the 90 °C processed material appears to show more C–O bonds, less C–O–H bonds, and possibly more C–Cl bonds, suggesting less of the PLA has been cleaved (because the O–C and O=C are closer to being stoichiometric) and less of the C–Cl moieties have been hydrolyzed to hydroxyls. This latter statement is also consistent with the IR spectra, which show much less evidence for hydroxyl stretches. The fraction of Ti–Cl bonds also appears to be lower at 90 °C processing. At both temperatures, XPS depth profiles (Figure S10) show infiltration through the entire depth of the material at even very short infiltration time (2 min), suggesting a reaction-limited process and that the depolymerization reaction is likely occurring throughout the entire bulk of the material, not just at the surface. Overall, these data appear to support that similar reactions are occurring at 135 °C, but just at a slower overall reaction rate, given the same reaction time. It is also possible that byproducts are not leaving because vapor pressures are significantly lower at this lower process temperature and/or the byproducts are larger oligomers with higher vapor pressures.

### Analysis of Byproducts and Byproduct Dissolution

**Figure 12:**
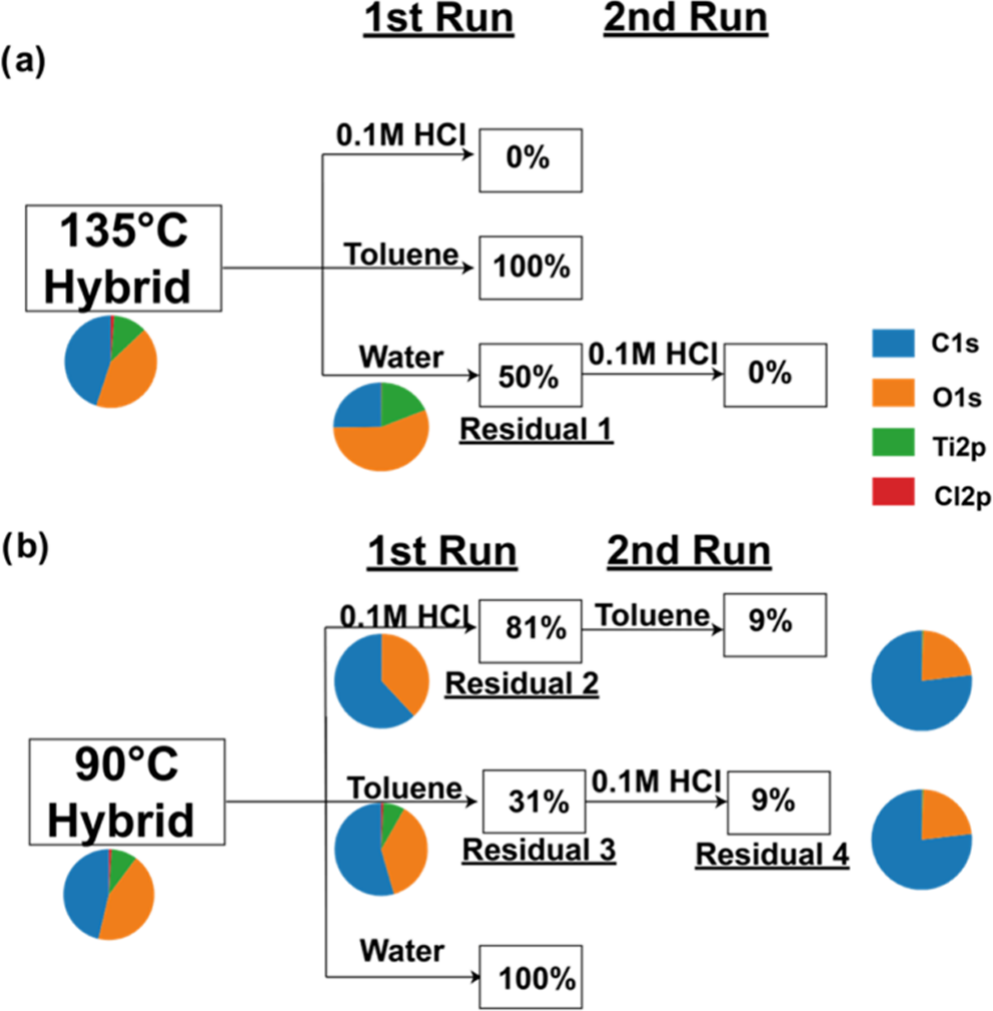
Analysis of PLA-TiO_*x*_ hybrids processed at (a) 135 °C and (b) 90 °C after immersion in various liquids (0.1 M HCl, toluene, and distilled water). Boxed percentages indicate remaining film thickness after 24 h of immersion. Pie charts show relative elemental compositions based on XPS analysis. As appropriate, some of the residuals were subsequently immersed in a second liquid (“2nd Run”).

To further understand the composition of the final infiltrated products and to determine if a combination of vapor processing and rinsing could be used to fully remove the polymer, we systematically exposed the films to various solvents including toluene, distilled water, and acidic water (pH = 1, 0.1 M HCl) for 24 h in various orders. Neat PLA dissolves in only toluene and does not dissolve in the other solvents. Amorphous ALD TiO_2_ thin films do not dissolve in any of the three solvents. We measured changes in film thickness and analyzed the elemental composition using XPS to understand the impact of solvent exposure on the hybrid material. Figure 12a,b depicts the thickness alterations in the PLA films post-infiltration at both 135 and 90 °C. We refer to any remaining material after a solvent exposure as a “residual”. The pie charts in Figure 12 show the relative elemental quantities determined by XPS for each of these residuals, and high-resolution XPS scans for each residual are given in Figure S11.

Figure 12a shows that the 135 °C TiO_*x*_ infiltrated PLA fully dissolves in the 0.1 M HCl, partially dissolves (50% decrease in thickness) in distilled water, and does not dissolve at all in toluene. This last observation, stability in a good solvent for the pure polymer, is similar to prior observations made for many other VPI hybrid materials.^1,30^ The residual after water immersion (“Residual 1”) has less carbon and more Ti and O than the original hybrid. This result suggests that the water is dissolving the organic material in this material, and we posit that these dissolved species are potentially partially depolymerized oligomers that are not volatile but are water-soluble, akin to oligomers of lactic acid or lactide. Interestingly, high-resolution scans in Figure S11 reveal that Residual 1 has nearly zero chloride emissions, suggesting that all chloride groups have been hydrolyzed by the water immersion. If this residual is subsequently immersed in 0.1 M HCl, the remaining residual is fully dissolved, suggesting the predominately inorganic species are susceptible to dissolution in an acidic solution; but these species are different from amorphous TiO_*x*_ given that ALD films of TiO_*x*_ were insoluble in this 0.1 M HCl solution.

Figure 12b shows that the 90 °C PLA-TiO_*x*_ hybrid does not dissolve at all in pure water, partially dissolves in an acidic solution (19% dissolved), and mostly dissolves in toluene (69% dissolved). The 0.1 M HCl immersion product (“Residual 2”) is particularly interesting as this process appears to remove all of the inorganic, leaving an organic composition comparable to pure PLA (60% C 1s, 40% O 1s). This PLA-like composition is further confirmed with the high-resolution XPS scans in Figure S11, which show nearly stoichiometric ratios of C–C, C–O, and C=O bonds in both the C 1s and O 1s spectra as expected for PLA.

In contrast, after toluene exposure (Residual 3), the material still contains a fully hybrid composition of carbon, oxygen, titanium, and chlorine, although it may be richer in organic content than the parent hybrid. This dissolution in toluene is interesting and consistent with prior observations where low reaction rates at low temperatures led to less chemical bond formation (cross-linking) and reduced resistance to dissolution in a good solvent for the pure polymer.^1^ Upon further exposure to 0.1 M HCl, most of this film is dissolved, although a residual organic is left (Residual 4). High-resolution scans in Figure S11 reveal that Residual 4 is largely hydrocarbon, which may be some byproduct of the decomposition and is consistent with the higher hydrocarbon surface content we had observed at the surface of the parent hybrids.

These experiments lead to a few conclusions. First, if complete PLA removal is desired, it is possible to first infiltrate at 135 °C and then immerse in 0.1 M HCl to completely remove the material. Complete etching with 90 °C infiltration does not appear possible under the conditions studied here; a thin (∼9%) residual hydrocarbon remains that is not easily removed with the solvents explored here. If conversion from PLA to a pure titanium oxide inorganic material is desired, infiltration at 135 °C followed by immersion in water can create a largely inorganic material. When we further combusted this material in a furnace at 700 °C for 1 h in air, we were able to transform the originally 40 nm PLA film into a 23 nm TiO_2_ film with a refractive index of 2.028.

## Conclusions

We previously demonstrated that vapor infiltration of TiCl_4_ into polymers can lead to the dealkylation of ester bonds. In this paper, we demonstrate that such chemistry can be applied to polymers with ester bonds in their main chain, for example, PLA, to depolymerize and etch them. Using QCM gravimetry, RGA mass spectrometry, FTIR spectroscopy, and XPS, we confirm that PLA depolymerizes during TiCl_4_ VPI. However, this process is dependent on both the temperature and the duration of precursor exposure. Depolymerization occurs readily at 135 °C with significant mass loss and film thickness reduction measured. In contrast, at 90 °C infiltration occurs, but thickness loss is minimal. Detailed chemical analysis suggests that similar depolymerization reactions are potentially occurring at 90 °C, but just much more slowly. The chemical assessment of mass loss, using FTIR, XPS, and RGA suggests that at higher temperatures, PLA is first depolymerizing to lactic acid or lactides and then decomposing even further to smaller byproducts (e.g., H_2_O, CO, CO_2_, CH_4_). This study advances our understanding of TiCl_4_ VPI into polymers containing ester functional groups and underscores the versatility of VPI to do both additive and subtractive processing.

## Supporting Information Available

Percent change in film thickness and refractive index before and after PLA infiltration; mass spectra of a TiCl_4_ dose when the walls are either passivated or not passivated; intensity of C 1s peaks with cluster ion etch time that shows significant reduction in C–C bond peak intensity; side-by-side comparison of TiCl_4_ infiltration at 135 and 90 °C: film thickness, *in situ* QCM, RGA mass spectra, and FTIR; additional RGA mass spectra of PLA powder exposed to TiCl_4_ and H_2_O at 135 and 90 °C processing temperature; XPS depth profiles of PLA thin films infiltrated with TiCl_4_ at 90 and 135 °C processing temperatures; and XPS spectra for residual materials derived from solubility tests. (PDF)
