# Supplementary material for: Depolymerization and Etching of Poly(lactic acid) via TiCl4 Vapor Phase Infiltration
Source: J Phys Chem C Nanomater Interfaces. 2024 Nov 13;128(47):20081–92. doi: 10.1021/acs.jpcc.4c04986 (PMC11613618; doi:10.1021/acs.jpcc.4c04986)
Supplement: Supplementary file 1 — jp4c04986_si_001.pdf [file jp4c04986_si_001.pdf]

Depolymerization and Etching of Poly(lactic acid) via $TiCl_4$ Vapor Phase Infiltration

*Shuaib A. Balogun [a] and Mark. D Losego [a,] **

a - School of Materials Science and Engineering, Georgia Institute of Technology, Atlanta, GA, 30318, USA

* Corresponding author : losego@gatech.edu

Supporting Information:

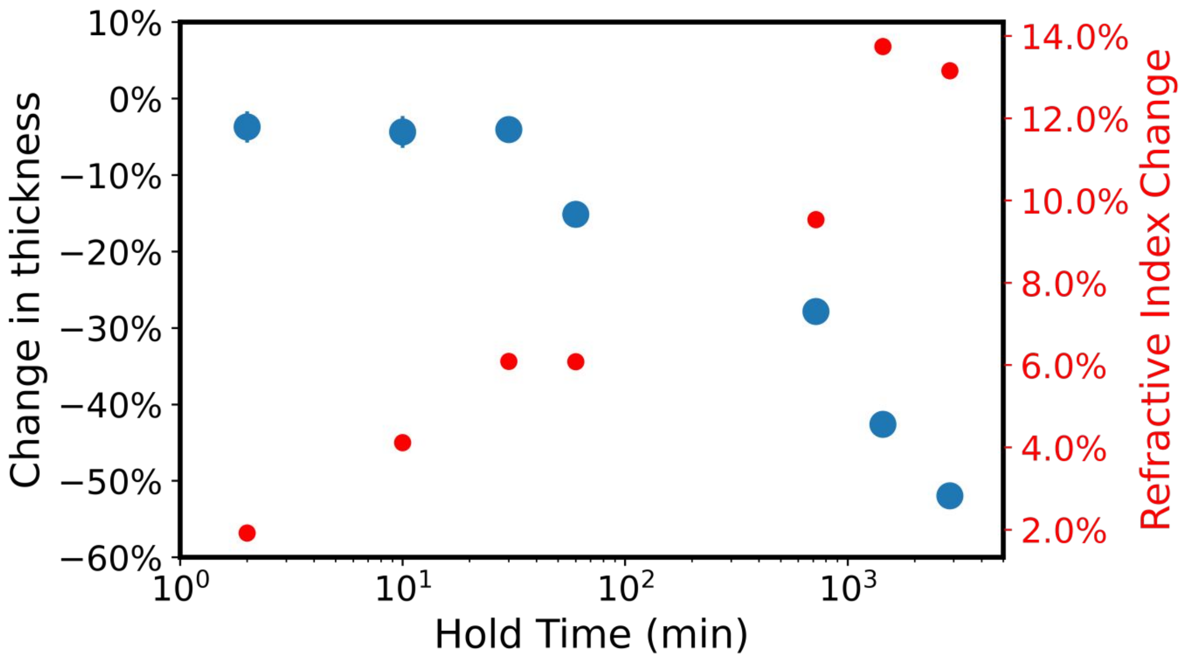

Figure S1: Percent change in film thickness and refractive index before and after infiltration as determined by spectroscopic ellipsometry for 200 nm PLA thin films infiltrated with $TiCl_4$ at 135 °C at varying precursor exposure times (0 – 48 h). Photographs show the visual change in film thickness.

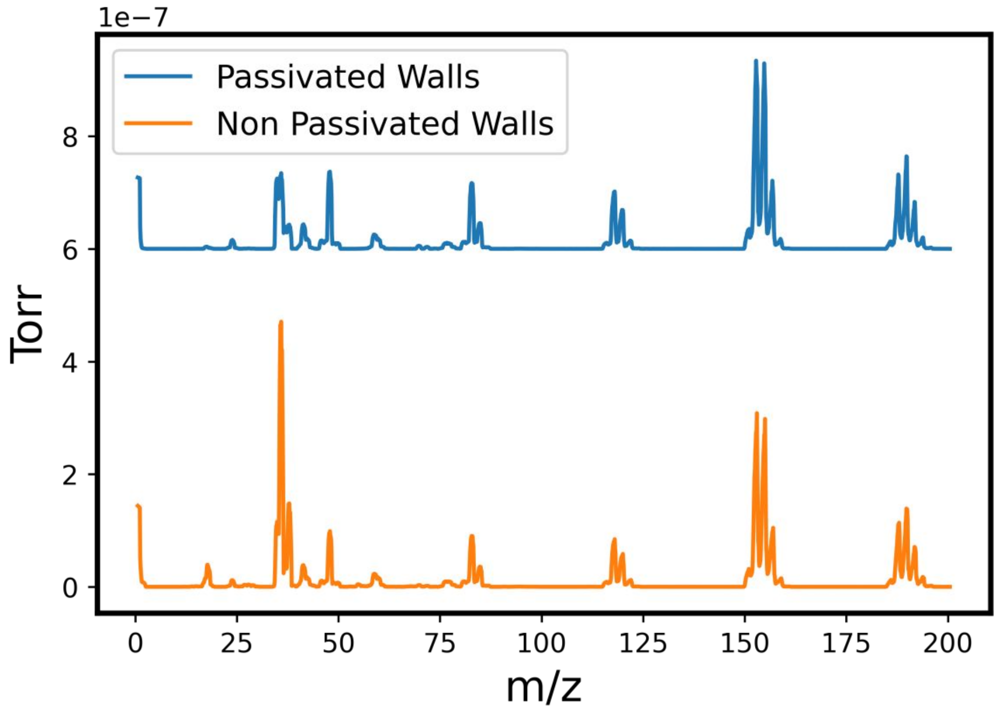

Figure S2: Mass spectra of a $TiCl_4$ dose when the walls are either passivated or not passivated.

The spectra for the “Passivated Walls” shows the ‘True’ mass spectra of $TiCl_4$. While the “Non-Passivated Walls” mass spectra shows a $TiCl_4$ dose where there are reactions between the $TiCl_4$ and residual water on the walls resulting in HCl formation.

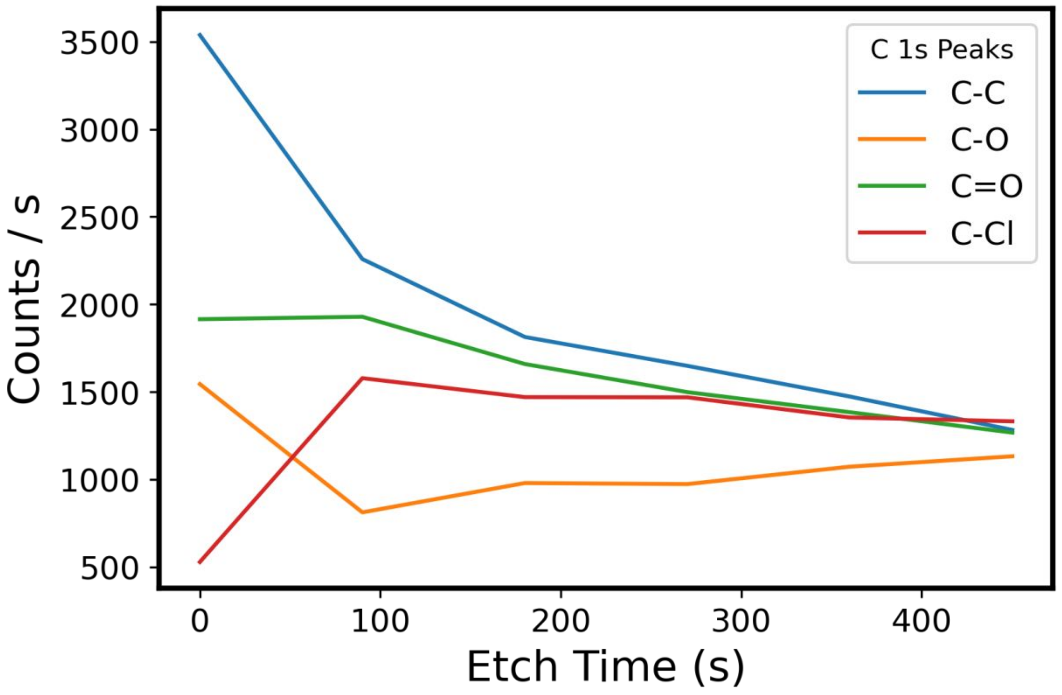

Figure S3: Intensity of C 1s peaks with cluster-ion etch time that shows significant reduction in C-C bond peak intensity corresponding to the removal of the adventitious carbon layer.

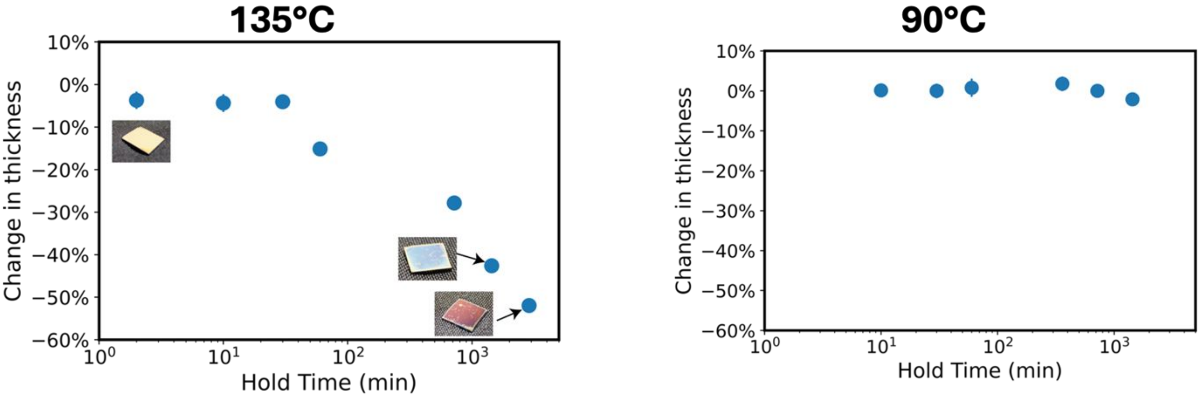

Figure S4: Side by side comparison of the change in thickness derived from spectroscopic ellipsometry of 200nm PLA thin films infiltrated with $TiCl_4$ at 135°C and 90 °C at various precursor exposure times (0 – 24h).

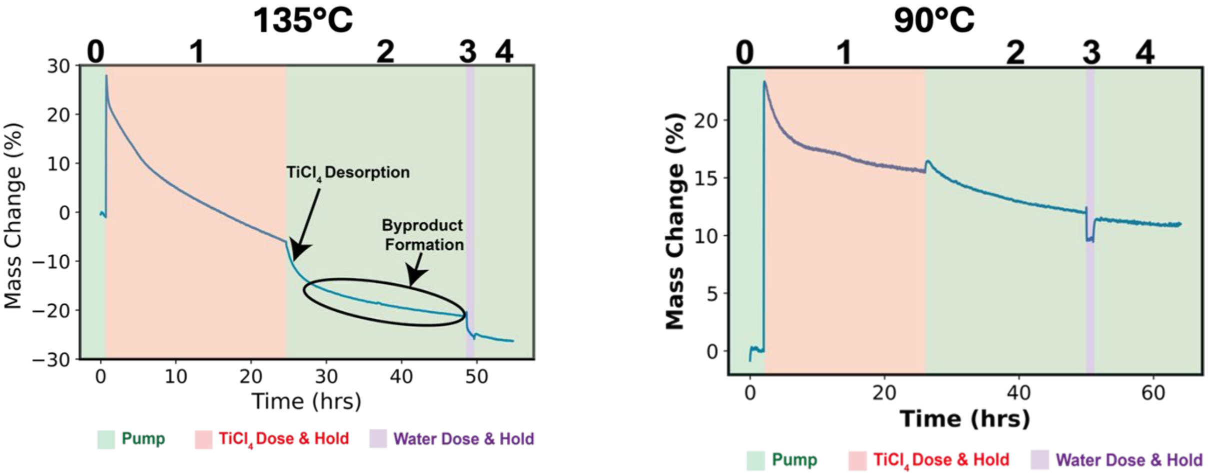

Figure S5: Side by side comparison of *In situ* QCM gravimetry of $TiCl_4$ infiltration into PLA at 135°C and 90 °C with 24 h of precursor exposure time.

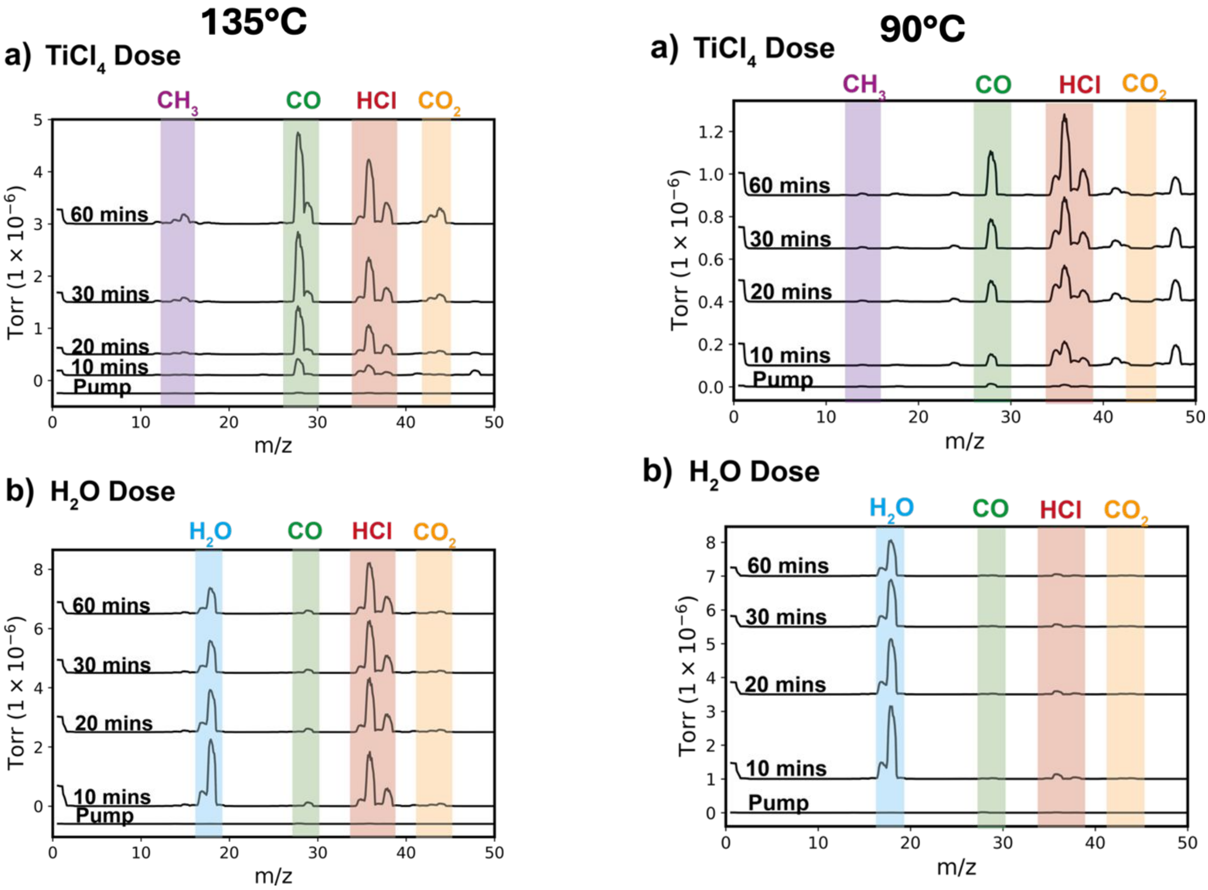
135°C
a) TiCl4 Dose
CH3
CO
HCl
CO2
60 mins
30 mins
20 mins
10 mins
Pump
Torr (1 × 10−6)
m/z
b) H2O Dose
H2O
CO
HCl
CO2
60 mins
30 mins
20 mins
10 mins
Pump
Torr (1 × 10−6)
m/z
90°C
a) TiCl4 Dose
CH3
CO
HCl
CO2
60 mins
30 mins
20 mins
10 mins
Pump
Torr (1 × 10−6)
m/z
b) H2O Dose
H2O
CO
HCl
CO2
60 mins
30 mins
20 mins
10 mins
Pump
Torr (1 × 10−6)
m/z

Figure S6: Side by side comparison of RGA mass spectra of the gas species above PLA powder exposed to a) $TiCl_4$ and b) H_2O at 135°C and 90 °C processing temperature at different exposure time

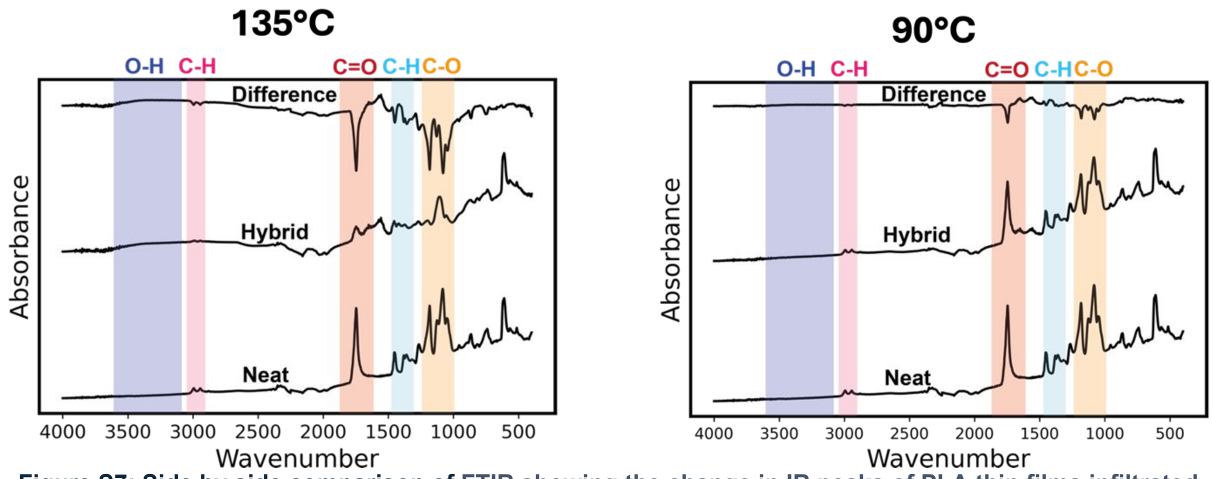

Figure S7: Side by side comparison of FTIR showing the change in IR peaks of PLA thin films infiltrated with $TiCl_4$ at 135°C and 90°C.

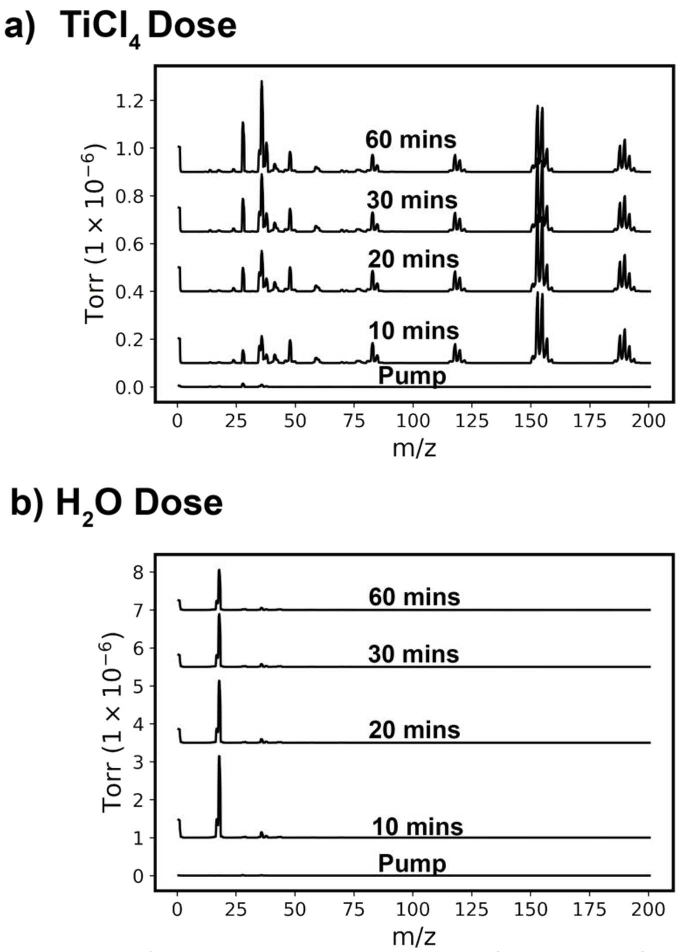

Figure S8: Full mass spectra of PLA powder exposed to a) $TiCl_4$ and b) H_2O at 90 °C processing temperature at different exposure times during the $TiCl_4$ dose and hold. The mass spectra data is segmented into distinct time intervals: 0 (pre-infiltration vacuum pumping), 10, 20, 30, and 60 minutes.

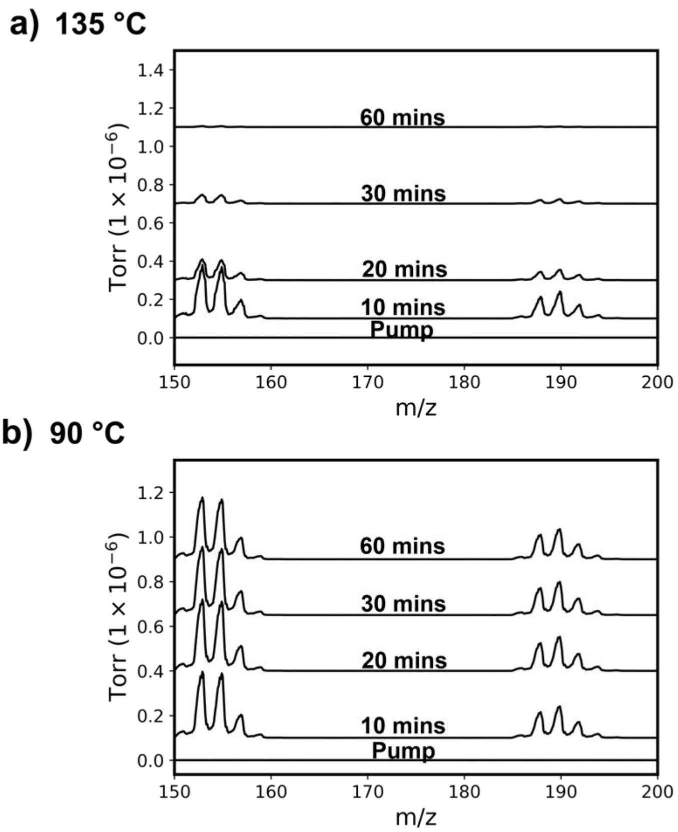

Figure S9: Mass spectra of PLA exposed to $TiCl_4$ at a)135 °C and b) 90 °C highlighting the $TiCl_4$ concentration in the gas phase as exposure time increases

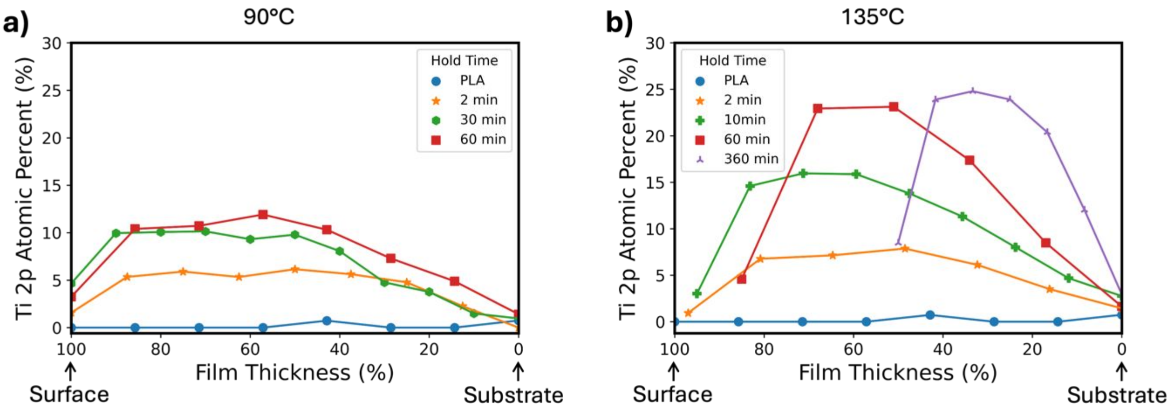

Figure S10: XPS depth profiles of PLA thin films infiltrated at a) 90°C and b) 135°C processing temperatures with varying $TiCl_4$ exposure times (0 min – 360 min). The surface of the thin films were shifted to represent the remaining thickness (shown in Figure 2 & 7) of the thin films at the varying exposure times.

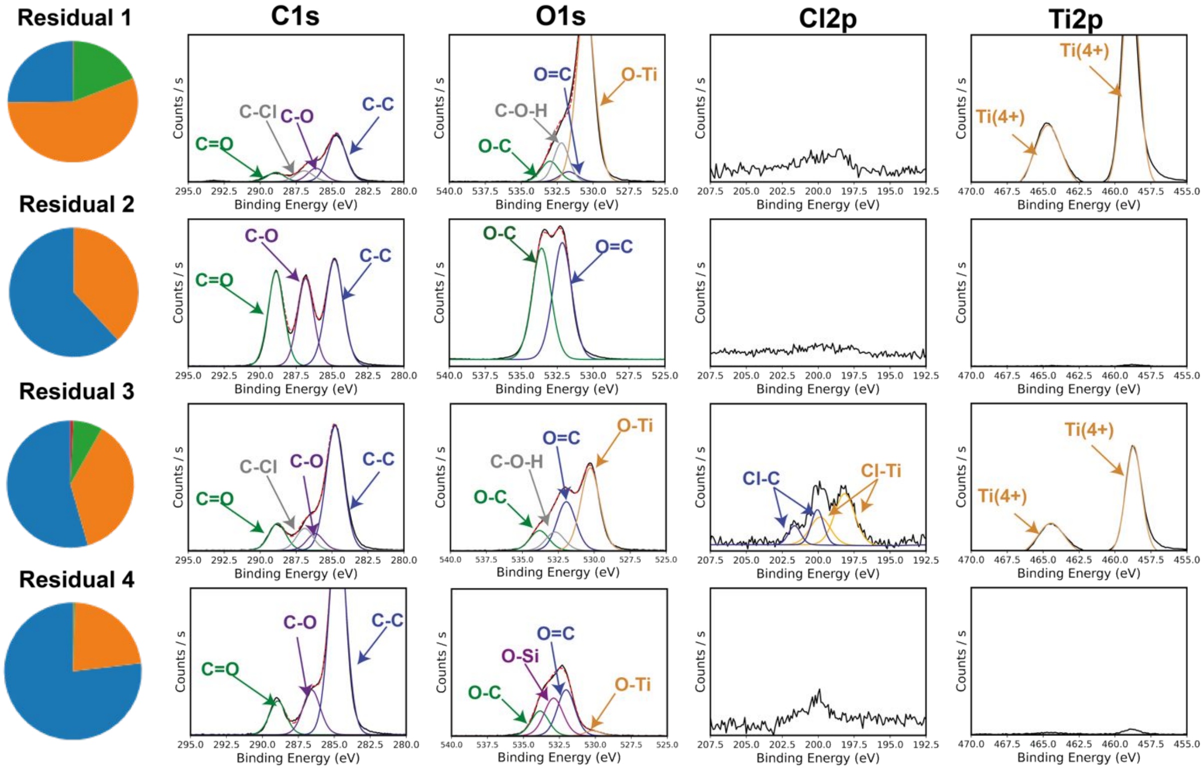

Figure S11: XPS Spectra for residual materials derived from solubility tests. The C 1s, O 1s, Cl 2p and Ti 2p spectra are shown for each residual film. Emission intensity axes (abscissas) are kept constant with the spectra in Figure 6. Deconvolutions are labelled for the C, O, Cl and Ti spectra
